# Photobiomodulation at 660 nm Alleviates Alzheimer’s Disease Pathology Through Amyloid-β Reduction and SIRT1 Upregulation in the Hippocampus of 5xFAD Mice

**DOI:** 10.3390/ijms26199569

**Published:** 2025-09-30

**Authors:** Tahsin Nairuz, Jin-Chul Heo, Hee-Jun Park, Jong-Ha Lee

**Affiliations:** Department of Biomedical Engineering, Keimyung University, Daegu 42601, Republic of Korea; tahsin.bmb@nstu.edu.bd (T.N.); washingbuffer@gmail.com (J.-C.H.); hjpark@kmu.ac.kr (H.-J.P.)

**Keywords:** Alzheimer’s disease, photobiomodulation therapy, 5xFAD mouse model, amyloid-β, SIRT1, hippocampus, Y-maze test

## Abstract

Alzheimer’s disease (AD) is a progressive neurodegenerative disorder characterized by amyloid-β (Aβ) accumulation, synaptic dysfunction, and cognitive decline. Current pharmacological treatments provide only symptomatic relief without altering disease progression. Photobiomodulation therapy (PBMT), a light-based intervention, has shown neuroprotective potential, although its exact neurobiological mechanisms in AD pathogenesis remain obscure. In this study, we investigated the effects of PBMT using a 660 nm wavelength light-emitting diode (LED) in 5xFAD transgenic mouse, a well-established model of early-onset AD. Mice were subjected to once daily PBMT sessions over a defined treatment period and outcomes were assessed through immunohistochemical analysis of hippocampal regions (CA1, CA2, CA3, and dentate gyrus) alongside behavioral testing using the Y-maze spontaneous alternation task. PBMT significantly reduced Aβ plaque load across hippocampal regions, accompanied by improved preservation of neuronal morphology. Furthermore, PBMT significantly upregulated SIRT1 expression, a critical regulator of synaptic plasticity and memory processes. Behaviorally, PBMT-treated mice displayed enhanced spatial working memory compared with controls, indicating a functional benefit linked to the observed molecular and structural changes. These findings suggest that 660 nm PBMT attenuates hallmark AD pathology, promotes neuroprotective pathways, and improves cognition, highlighting its potential as a disease-modifying therapy that warrants further preclinical and clinical investigation.

## 1. Introduction

Alzheimer’s disease (AD), a severe neurodegenerative disorder and the predominant manifestation of dementia, afflicts over 50 million individuals aged 65 and above worldwide, amplifying pressures on global healthcare systems [1]. The disease is primarily defined by a gradual decline in cognitive functions, particularly memory impairment, which worsens over time [2]. Histopathologically, AD is characterized by extracellular deposition of insoluble fibrillar amyloid-β peptides forming senile plaques and intraneuronal aggregation of hyperphosphorylated tau as neurofibrillary tangles [3]. These hallmark abnormalities are further accompanied by synaptic dysfunction, extensive neuronal loss, and disrupted metal-ion homeostasis [4]. Despite significant advancements in research, the precise molecular mechanisms governing AD pathogenesis remain elusive.

The amyloid-β (Aβ) cascade hypothesis remains a cornerstone in AD research, positing Aβ overproduction as a pivotal initiator of pathophysiological processes through its disruption of cellular metabolism and initiation of downstream neurodegenerative processes [5]. Intraneuronal Aβ accumulation, particularly in brain regions vulnerable to AD, impairs synaptic plasticity, mitochondrial dysfunction, axonal degeneration, and DNA instability [6]. Concurrently, the extensive deposition of amyloid plaques and neurofibrillary tangles exacerbate astrogliosis, neuronal loss, synaptic disruption, and vascular abnormalities [7]. Despite the prolonged emphasis on therapeutic strategies aimed at blocking extracellular Aβ deposition and clearing plaques to alleviate symptoms, restoring functional integrity in damaged neurons remains a significant challenge. Consequently, emerging therapeutic approaches advocate for integrative therapies that not only mitigate Aβ pathology but also restore neuronal function to effectively combat AD progression.

Sirtuins are a family of NAD+-dependent protein deacetylases that are recognized for their protective roles in mitigating age-related conditions including diabetes, cancer, heart disease, and neurological disorders [8]. The mammalian ortholog of yeast SIR2, Sirtuin 1 (SIRT1), is one of the seven mammalian sirtuins that is particularly crucial for ameliorating neurodegenerative pathologies by supporting cognitive function and neurogenesis, enhancing neurite outgrowth, preserving synaptic plasticity, and promoting neuronal survival [9]. Prior research has demonstrated that SIRT1 mitigates Aβ-induced toxicity and cognitive impairments in animal models, primarily through reducing Aβ production in neurons and suppressing inflammatory responses in neuroglial cells [10,11]. Another study revealed that SIRT1 knock-out (KO) mice exhibited marked deficits in both hippocampus-dependent short- and long-term memory [12]. Importantly, recent studies have also shown that PBMT can modulate SIRT1 signaling pathways, thereby linking PBMT with reductions in Aβ burden and improved neuronal survival. Zhang et al. (2020) demonstrated that PBMT activates the PKA/SIRT1 pathway, leading to decreased Aβ levels [13], while Huang and Hamblin (2024) highlighted SIRT1 as a critical mediator of PBMT’s neuroprotective effects [14]. Collectively, this strong mechanistic and experimental evidence underscores SIRT1 as one of the most biologically meaningful and experimentally validated molecular targets for understanding how PBMT confers neuroprotection in Alzheimer’s disease.

Photobiomodulation (PBM), delivered through light-emitting diodes (LEDs) or low-level laser therapy (LLLT), has emerged as a novel, non-invasive, drug-free therapeutic modality in health management, dermatology, and psychological interventions, appreciated for its safety, cost effectiveness, accessibility, and ease of application [15]. PBM has demonstrated efficacy in treating neurological conditions, including traumatic brain injury (TBI), stroke, and neurodegenerative diseases [16]. Empirical evidence suggests that PBMT exerts potent neuroprotective effects in AD by mitigating dendritic atrophy, reducing neuronal loss, and attenuating Aβ-triggered neuronal apoptosis [13,14]. Furthermore, PBM’s cognitive benefits extend to healthy subjects and animal models, with studies reporting that LED irradiation at 1072 nm improved spatial navigation in a 3D maze, and 660 nm LED exposure enhanced extinction of conditioned fear and prevented its reinstatement [17,18]. These findings underscore PBM’s potential in combating neurodegenerative conditions in AD, where its capacity to reduce neuronal loss and promote synaptic resilience may slow disease progression. Supporting this, repeated transcranial laser stimulation in AD mouse models resulted in significant neurological improvement compared to non-treated controls [19,20]. Taken together, these mechanistic insights underscore PBM’s therapeutic promise in mitigating neurodegeneration, preserving cognitive function, and potentially altering the progression of AD.

While PBMT is widely regarded as a non-invasive therapeutic modality, its application in small animal models poses significant challenges due to limited light penetration caused by the relative thickness and scattering properties of the scalp and skull. Shaw and colleagues reported that nearly 90% of the near-infrared signal is lost in mice following transcranial LED application, highlighting substantial attenuation [21]. Similar findings have been observed in human cadaveric specimens, emphasizing the broader translational challenge [22]. Furthermore, De Taboada and colleagues estimated over 99% power density loss at a depth of 18 mm in rats [23], while Abdo and colleagues noted approximately 90% signal attenuation just 1 mm below the cortical surface [24]. These significant reductions in light transmission suggest that fully transcranial PBM application may be insufficient for achieving therapeutic efficacy in deep brain regions and may not support its use as a reliable long-term treatment. Consequently, partially invasive or intracranial PBM methods are frequently adopted in preclinical studies to bypass these limitations, enabling more accurate and reproducible light delivery to targeted brain areas. For instance, Darlot et al. utilized an intracranial approach in a primate model, and Moro et al. employed direct intracranial light delivery in a mouse model of Parkinson’s disease, demonstrating its neuroprotective effects on targeted brain regions [25,26]. While such methods do not represent the typical non-invasive applications seen in clinical settings, they are essential in animal models for overcoming anatomical constraints and accurately assessing the biological efficacy of PBMT.

In this study, we explored the effects of LED-applied PBM therapy on AD neuropathology in 5xFAD transgenic mice, focusing on the impact of both the presence and timing of PBM administration. We preferred to use LED instead of a laser in this experiment, as low-power LEDs offer several advantages, including affordability, portability, and ease of use. Moreover, a previous study demonstrated that the LED generated no detectable heat across both the implant and cranium, confirming its safety and suitability for extended applications lasting up to six days [25]. Given the critical role of the hippocampus in memory formation and cognitive function, we specifically examined PBM-induced changes within this region, which undergoes significant structural, morphological, and electrophysiological alterations during AD [27]. These pathological alterations are notably evident in specific hippocampal regions CA1, CA2, CA3, and the dentate gyrus (DG), each characterized by distinct anatomical features and functional specializations. To evaluate the cumulative effects of treatment, PBMT was administered once daily for 14 consecutive days. Our primary objective was to evaluate the effects of PBM on Aβ plaque load and SIRT1 expression in the hippocampus of 5xFAD AD mouse model. We also explored the impact of PBM in the prevention and/or recovery of cognitive deficits in 5xFAD mice through behavioral assessment. Therefore, the significance of this study lies in its demonstration of PBM as a novel therapeutic strategy for slowing AD progression, primarily by targeting Aβ production and enhancing SIRT1-mediated neuroprotection.

## 2. Results

### 2.1. PBMT at 660 nm Restores Cellular Morphology of 5xFAD Mice Hippocampus

**Figure 1:**
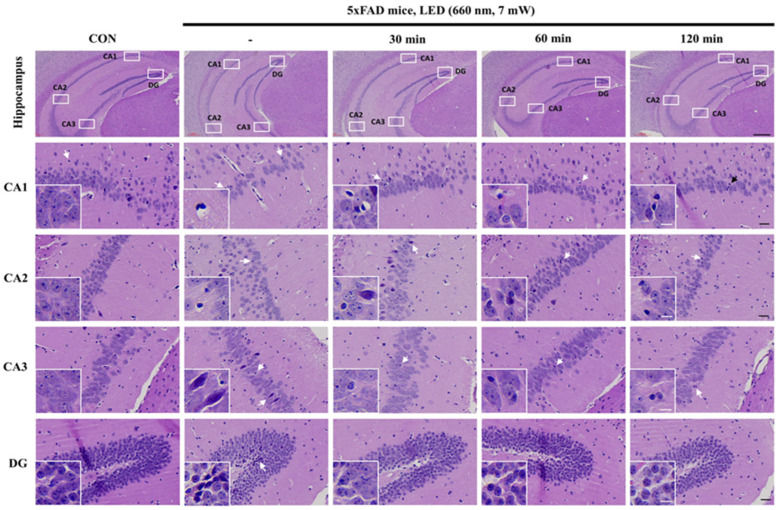
Representative H&E-stained hippocampal sections (CA1, CA2, CA3, and DG) in control (CON) and 5xFAD mice after PBMT (660 nm LED, administered once daily for 14 consecutive days) for 30, 60, and 120 min. The images illustrate structural differences in hippocampal morphology across treatment groups, with insets providing higher magnification views of cellular details. Arrows indicate regions of interest, highlighting potential changes in neuronal integrity and cellular organization. Scale bars: 300 µm (overview), 30 µm (magnified sections), and 10 µm (insets).

Histological variations, such as cellular morphology, tissue integrity, and potential structural changes in response to experimental conditions, were identified though H&E immunostaining images of CA1, CA2, CA3, and DG hippocampus regions of control and 5xFAD mice. The framed regions are presented at higher magnification in Figure 1 to compare morphological features between control and 5xFAD mice subjected to LED irradiation for 30, 60, and 120 min respectively.

In the control group, the hippocampal CA1, CA2, and CA3 regions exhibited a well-organized pyramidal cell layer with densely packed, clear nuclei, and intact cytoplasmic structures, with no signs of cellular disarray, degeneration, or inflammatory infiltration. Similarly, the granule cell layer of DG region was densely packed exhibiting a uniform arrangement and well-defined nuclei. The granule cells maintained their characteristic compact organization, with a clear distinction between the molecular and polymorphic layers. The absence of nuclear fragmentation, cytoplasmic vacuolization, or lymphocytic infiltration further indicated preserved cellular integrity in the hippocampal regions of the control group.

However, the hippocampal regions of 5xFAD mice exhibited significant pyramidal and granule cell loss, disorganized cell layers, and substantial lymphocyte infiltration. The CA1, CA2, and CA3 pyramidal cell layers were atrophied, with small, damaged cells showing condensed nuclei, dark cytoplasm, and disrupted morphology. Some cells lost their pyramidal shape, while surrounding cells appeared atrophic. In DG region, the granule cell layer was highly disorganized, with degenerated granule cells displaying vacuolation and dark, condensed nuclei, indicating severe structural deterioration.

Following LED therapy, the histopathological alterations observed in 5xFAD mice demonstrated notable amelioration over time, signifying a restorative effect on hippocampal structure. The pyramidal layers in CA1 and CA3 maintained relatively normal thickness, with vesicular nuclei and pale basophilic cytoplasm. Additionally, DG region retained its structural integrity, with densely packed granule cells exhibiting round, pale vesicular nuclei. These findings indicate the efficacy of PBM therapy at 660 nm in mitigating hippocampal neurodegeneration and preserving neural integrity in 5xFAD mice.

### 2.2. PBMT at 660 nm Reduces Aβ Plaque Load in 5xFAD Mice Hippocampus

Since Aβ plaques accumulation leads to cognitive impairment in AD, we investigated whether PBM treatment had any impact on amyloid plaque load. In 5xFAD mice, intraneuronal accumulation of β-amyloid is observed as early as 1.5 months, with extracellular plaque formation emerging by 2 months, mainly in the subiculum, cortex, and hippocampus [28,29]. The 5xFAD mice used in this experiment were 6–7 weeks old, meaning a significant number of amyloid plaques were likely visible in the hippocampus. Immunohistochemistry using anti-Aβ1-40 and anti-Aβ1-42 antibodies was performed in the CA1, CA2, CA3, and DG regions of the hippocampus to visualize the plaque load in control and 5xFAD mice.

**Figure 2:**
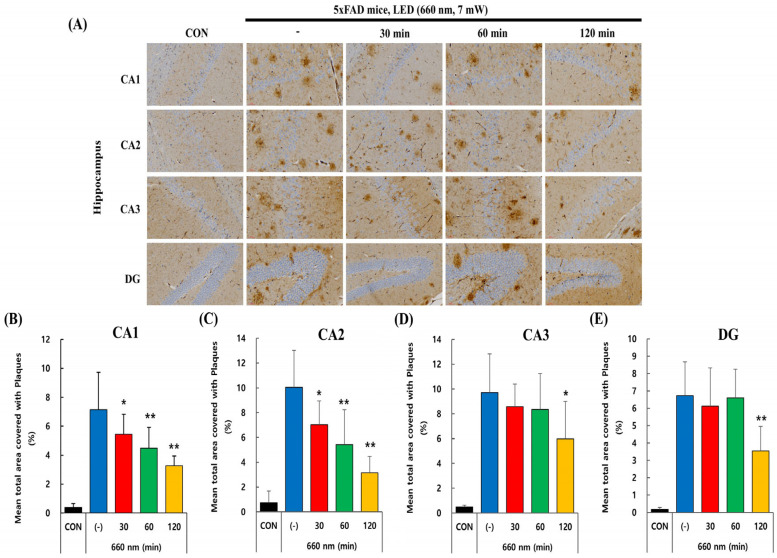
PBMT at 660 nm reduces Aβ1-40 plaque burden in 5xFAD mice hippocampus. (**A**) Representative immunohistochemical images of Aβ plaques in hippocampal regions (CA1, CA2, CA3, and DG) after LED (660 nm, 7 mW) treatment for 30, 60, and 120 min. Scale bar: 30 µm. (**B**) Quantitative analysis of plaque-covered area (%) in CA1, (**C**) CA2, (**D**) CA3, and (**E**) DG, showing a significant reduction in Aβ plaques with prolonged PBMT duration. Quantified data are presented as the mean ± SEM. * *p* < 0.05, ** *p* < 0.01 vs. untreated 5xFAD mice.

Figure 2A–E illustrates the effect of PBM treatment at 660 nm on Aβ1-40 plaque load in different hippocampal regions (CA1, CA2, CA3, and DG). The control group showed minimal plaque accumulation, while the untreated 5xFAD mice group exhibited a substantial increase in plaque deposition, confirming the pathological amyloid buildup characteristic of 5xFAD mice. The graphs in Figure 2 indicate that plaque load was significantly reduced in CA1 and CA2 regions after PBM treatment, with the most notable decline observed at 60 and 120 min (*p* < 0.01). CA3 showed a moderate reduction, with a significant decrease at 120 min (*p* < 0.05). The DG region exhibited the least response to PBM treatment, with plaque load remaining relatively stable at 30 and 60 min but showing a significant reduction at 120 min (*p* < 0.01). Overall, longer LED exposure (120 min) led to a greater reduction in amyloid plaques across all regions, with CA1 and CA2 being the most responsive to treatment.

**Figure 3:**
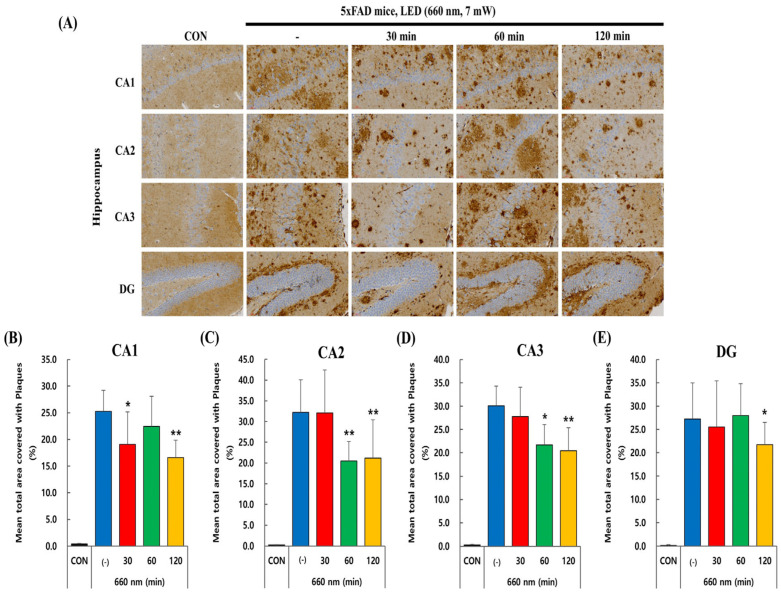
PBMT at 660 nm reduces Aβ1-42 plaque burden in 5xFAD mice hippocampus. (**A**) Representative immunohistochemical images of Aβ plaques in hippocampal regions (CA1, CA2, CA3, and DG) after LED (660 nm, 7 mW) treatment for 30, 60, and 120 min. Scale bar: 30 µm. (**B**) Quantitative analysis of plaque-covered area (%) in CA1, (**C**) CA2, (**D**) CA3, and (**E**) DG, showing a significant reduction in Aβ plaques with prolonged PBMT duration. Quantified data are presented as the mean ± SEM. * *p* < 0.05, ** *p* < 0.01 vs. untreated 5xFAD mice.

Figure 3A–E also represents Aβ1-42 plaque load reduction in the CA1, CA2, CA3, and DG regions following PBMT at 660 nm. In CA1 region, the untreated group showed high plaque accumulation, which significantly decreased at 30 (*p* < 0.05) and 120 min (*p* < 0.01). A similar trend was seen in CA2, where plaque load also decreased significantly at 60 and 120 min (*p* < 0.01), indicating a strong response to LED exposure. CA3 exhibited a significant decrease in plaque load at 60 min (*p* < 0.05) and a more pronounced effect at 120 min (*p* < 0.01), suggesting a delayed but effective response. The DG region demonstrated the least reduction, with minimal changes at 30 and 60 min but a significant decrease only at 120 min (*p* < 0.05). Overall, the data suggest that longer LED exposure, particularly 120 min, is more effective in reducing amyloid plaques across all hippocampal regions.

### 2.3. PBMT at 660 nm Modulates SIRT1 Expression in 5xFAD Mice Hippocampus

Recent studies highlight the crucial role of SIRT1 in protecting neurons in both cultured cells and animal models [30,31]. In the hippocampus, SIRT1 is predominantly localized within the nuclei of pyramidal and granule neurons, where it is critically involved in modulating synaptic plasticity and cognitive processes. We examined SIRT1 expression in the hippocampus, using mouse-specific anti-SIRT1 antibody to determine the impact of PBM therapy on SIRT1 levels in the CA1, CA2, CA3, and DG regions of control and 5xFAD mice.

**Figure 4:**
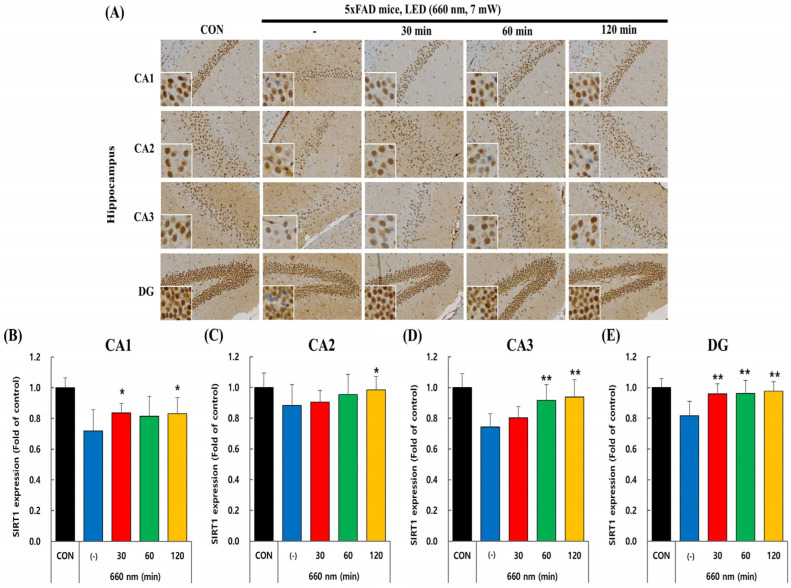
Effect of PBMT at 660 nm on SIRT1 expression in 5xFAD mice hippocampus. (**A**) Representative immunohistochemical images showing SIRT1 expression in hippocampal regions (CA1, CA2, CA3, and DG) following LED (660 nm, 7 mW) treatment for 30, 60, and 120 min. Scale bar: 30 µm and 10 µm. (**B**) Quantification of SIRT1 expression levels in CA1, (**C**) CA2, (**D**) CA3, and (**E**) DG, indicating a significant SIRT1 upregulation with increasing PBMT duration. Quantified data are presented as the mean ± SEM. * *p* < 0.05, ** *p* < 0.01 vs. untreated 5xFAD mice.

Figure 4A–E illustrates the effect of PBM at 660 nm on SIRT1 expression in CA1, CA2, CA3, and DG hippocampal regions. The untreated group exhibits lower SIRT1 expression than the control, indicating AD-related downregulation. In CA1 and CA2, PBM at 30 and 120 min led to a moderate but significant increase (*p* < 0.05), while CA3 exhibited a stronger response with 60 and 120 min significantly upregulating SIRT1 (*p* < 0.01). The DG region showed the most consistent effect, with all PBM durations (30, 60, and 120 min) significantly increasing SIRT1 expression (*p* < 0.01), indicating that DG is the most responsive to PBM treatment. These findings imply that PBMT has a modulatory effect on SIRT1 expression, potentially contributing to neuroprotection in AD-affected hippocampal regions.

### 2.4. PBMT at 660 nm Improves Memory Deficits in 5xFAD Mice

**Figure 5:**
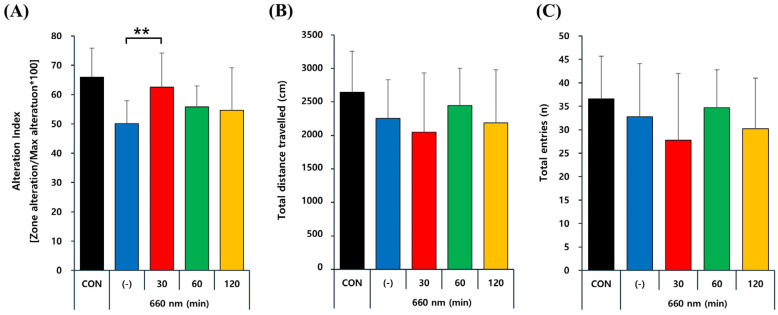
Effect of PBMT at 660 nm for 30, 60, and 120 min on spatial memory in 5xFAD mice assessed by the Y-maze test. (**A**) PBMT improves spatial working memory (alternation index). (**B**) No significant differences in locomotor activity (total distance traveled). (**C**) Comparable exploratory behavior (total entries). Quantified data are presented as the mean ± SEM. ** *p* < 0.01 vs. untreated 5xFAD mice.

Excessive Aβ accumulation is a critical factor in AD pathogenesis, strongly linked to spatial learning and memory impairments [7]. To explore the therapeutic potential of PBM, its effects on Aβ-induced cognitive deficits were evaluated using Y-maze test. Figure 5 represents Y-maze test results following PBM treatment at 660 nm in control and 5xFAD mice, assessing spatial working memory through the alteration index (A), total distance traveled (B), and total entries (C). The alteration index, which measures working memory performance, is significantly lower in untreated 5xFAD mice (-) compared to controls but shows a notable improvement with 30 min of PBMT (*p* < 0.01), while 60 and 120 min show mild but non-significant effects. In contrast, total distance traveled and total entries, indicators of locomotor activity and exploratory behavior, remain unchanged across all groups, indicating that the cognitive benefits of PBM are not due to increased movement but rather enhanced memory function. These findings suggest that PBMT has a beneficial effect on spatial working memory in 5xFAD mice, potentially counteracting cognitive deficits associated with AD.

## 3. Discussion

A primary avenue in AD research has been the development of pharmacological interventions targeting amyloid-β pathology by modulating its aggregation, clearance, or structural stability. Currently, the most widely used pharmacotherapies, including N-methyl-D-aspartate (NMDA) receptor antagonists and acetylcholinesterase (AChE) inhibitors, provide only symptomatic relief without modifying the underlying disease trajectory [32]. Given the multifactorial etiology of AD, these treatments fail to halt or decelerate disease progression, thereby underscoring an urgent need for innovative therapeutic strategies capable of addressing the neurodegenerative mechanisms driving AD pathology. Photobiomodulation therapy, a nonthermal irradiation technique employing visible to near-infrared light, has demonstrated efficacy in reducing pain and inflammation across diverse pathological conditions [33]. Elucidating the molecular mechanisms and functional significance of PBMT in neurodegeneration could provide critical insights into its capacity to address both the pathological and symptomatic dimensions of AD.

The present study investigated the efficacy of PBMT as a preventive strategy in a transgenic AD mouse model, with a particular focus on the impact of treatment duration. We utilized 5xFAD transgenic mice that coexpresses five familial AD mutations in amyloid precursor protein (APP) and presenilin 1 (PS1), leading to accelerated Aβ deposition and recapitulating key neuropathological and behavioral features of AD, including working memory impairment, disinhibitory behavioral tendencies with reduced anxiety, extensive amyloid plaque formation, and selective neuronal loss [34,35]. However, this APP/PS1 model does not exhibit neurofibrillary tangle formation, representing a plaque-predominant AD phenotype observed in autopsy studies [36]. Aβ pathology in 5xFAD mice is evident as early as 2 months of age, with a rapid accumulation of plaques observed by 4 months, initially concentrated in the cortical regions before extending to the hippocampus. By 6 months of age, these mice exhibit a pronounced and widespread amyloid burden affecting both cortical and hippocampal structures [34].

Regarding light penetration properties, near-infrared wavelengths such as 810–1072 nm penetrate brain tissue more effectively due to lower scattering and absorption and are therefore often considered advantageous for deep-brain stimulation. In contrast, 660 nm light has inherently lower intracranial penetration because of higher absorption by endogenous chromophores such as hemoglobin and melanin, which limit the amount of light reaching deeper structures [37]. Despite this limitation, 660 nm was selected in this study because of its well-documented neuroprotective efficacy in AD models and the practical advantages of LED devices at this wavelength, which are cost-effective, portable, generate negligible heat, and have been proven safe for repeated use in small-animal studies. Previous preclinical work has shown that 660 nm PBMT reduces amyloid-β burden and activates protective signaling pathways such as PKA/SIRT1 [13]. In the present study, we adopted a surgical method involving scalp removal and burr hole creation in 5xFAD mice to allow direct intracranial LED placement, thereby overcoming the limited penetration of 660 nm light. This approach enabled consistent photonic delivery to deep brain regions and ensured reliable hippocampal exposure while leveraging the established biological activity of 660 nm PBMT. Importantly, this partially invasive method aligns with previous preclinical studies in AD mouse models that have used similar techniques to enhance light transmission efficiency and target deep brain structures [19,38]. Although this method introduces a degree of invasiveness, it was deemed necessary to optimize energy delivery and reduce light scattering, especially considering the low power (7 mW) and wide beam divergence (120°) of the applied LED. To control for potential confounders such as surgical trauma or inflammatory response, sham-operated mice received identical treatment protocols without active irradiation. Despite operating below the established photothermal damage threshold, we monitored cranial tissues during and after LED application and found no visible signs of thermal damage, such as discoloration, edema, or inflammation, confirming the procedural safety. Additionally, histological analyses of hippocampal sections revealed no evidence of heat-induced cellular damage or inflammation.

Given the central role of amyloid-β in AD pathology, we evaluated Aβ plaque burden as a key biomarker in this study. To assess the preventive potential of PBMT, we initiated treatment in 5xFAD transgenic mice at around 1.5–2 months of age, coinciding with the earliest stages of Aβ deposition. This approach enabled us to systematically examine the capacity of PBMT to modulate Aβ accumulation and intervene before substantial neuropathological alterations occurred. Notably, previous research has indicated that PBM exhibits diminished efficacy when administered after the onset of established pathology [39]. As anticipated, in our experiment PBMT significantly reduced Aβ plaque load in the hippocampus in a time-dependent manner, with prolonged exposure amplifying its efficacy (Figure 2 and Figure 3). These reductions were most evident in CA1 and CA2, while the dentate gyrus showed comparatively weaker changes, consistent with evidence that Aβ pathology is unevenly distributed across hippocampal subregions, with CA1 and CA3 being more vulnerable to early accumulation [40,41]. Such region-specific effects likely reflect intrinsic differences in pathological progression and responsiveness to treatment. Our findings align with previous studies demonstrating the efficacy of PBM therapy in reducing Aβ pathology in different AD models [38,39]. Similarly, prior research had shown that prolonged exposure to PBMT at 632.8 nm markedly reduced Aβ accumulation and amyloid plaque load in vivo in APP/PS1 mice hippocampus [13]. Collectively, these results reinforce the use of Aβ burden as a biomarker and suggest that PBMT may preferentially mitigate pathology in hippocampal regions most vulnerable to early amyloid deposition.

Mechanistically, this reduction in Aβ burden following PBMT treatment is primarily driven by its regulatory effects on Aβ production and processing. In AD, excessive Aβ accumulation results from both increased synthesis and impaired catabolism, with deficits in key Aβ-degrading enzymes, such as neprilysin (NEP) and insulin-degrading enzyme (IDE), playing a crucial role in its pathological buildup [42]. Although APP/PS1 mice showed decreased IDE expression, PBMT treatment did not produce a significant change in the protein levels of enzymes responsible for Aβ degradation, indicating that clearance mechanisms remained largely unchanged. Instead, PBMT influenced APP processing by upregulating ADAM10, the primary α-secretase in the non-amyloidogenic pathway, while downregulating BACE1, the β-secretase responsible for initiating Aβ synthesis [13]. This shift in APP metabolism reduced Aβ production by favoring α-secretase-mediated cleavage over β-secretase activity, suggesting that PBMT mitigates Aβ accumulation by modulating its biosynthetic pathway rather than enhancing its enzymatic degradation.

SIRT1 has emerged as a key regulator of neuronal resilience, where its activation is critical for maintaining synaptic plasticity and cognitive function, while its dysfunction precipitates neuronal deficits [43]. In AD, a significant decline in SIRT1 expression was observed, correlating with increased amyloid-β production. Conversely, upregulation of SIRT1 has been shown to counteract Aβ accumulation, emphasizing its fundamental role in modulating amyloidogenic pathways [44]. At the molecular level, SIRT1 exerts its neuroprotective effects through the deacetylation of RARβ, thereby enhancing ADAM10 expression and redirecting APP processing toward the non-amyloidogenic pathway, ultimately suppressing Aβ synthesis in vitro in embryonic neurons from Tg2576 mice [10,45]. Additionally, SIRT1 activation promotes PGC-1α signaling, which in turn suppresses BACE1 expression, further attenuating Aβ production [46,47]. These mechanistic insights suggest a novel regulatory axis, wherein the modulation of ADAM10 and BACE1 by PBMT may stem from SIRT1-driven deacetylation processes, reinforcing the hypothesis that SIRT1 serves as a central molecular target in PBMT-mediated neuroprotection. Our findings align with this premise, as prolonged PBMT led to a significant upregulation of SIRT1 expression in the hippocampus of 5xFAD mice (Figure 4). This elevation in SIRT1 levels was accompanied by a substantial reduction in Aβ accumulation, suggesting that PBMT-mediated SIRT1 activation plays a crucial role in mitigating amyloid pathology. These results indicate that the neuroprotective effects of PBMT may be, in part, attributed to SIRT1-mediated modulation of Aβ metabolism, thereby facilitating the preservation of hippocampal integrity in AD-affected regions.

Furthermore, this experiment demonstrated a significant improvement in working memory performance in 5xFAD mice, as reflected by an increased alternation index in the Y-maze test following PBMT (Figure 5). This finding is consistent with previous research demonstrating that PBM therapy during the initial phase of amyloid accumulation effectively improves memory deficits and mitigates anxiety-related behaviors in AD mouse model [39]. Moreover, previous research has established the critical function of SIRT1 in facilitating the acquisition and consolidation of both short-term and long-term hippocampus-dependent memories, while preserving baseline synaptic function, the structural integrity of CA1 dendrites, and synaptic protein levels [12]. Given that our study demonstrates a significant increase in SIRT1 expression and a concurrent reduction in Aβ plaque burden following PBMT, it is plausible that these molecular alterations contribute to the observed cognitive improvements in 5xFAD mice. Interestingly, while PBMT led to a pronounced, duration-dependent reduction in Aβ plaque burden and upregulation of SIRT1 expression, a significant improvement in spatial working memory was observed only in the 30-min treatment group. This apparent discrepancy may be explained by the biphasic dose–response relationship commonly observed in PBMT [48], where low to moderate energy doses elicit optimal therapeutic effects, and higher doses may result in a plateau or even diminished functional outcomes. It is possible that extended PBMT durations, while enhancing pathological clearance, may exceed the optimal threshold for neurocognitive modulation, leading to compensatory effects or metabolic fatigue that obscure behavioral gains. Additionally, working memory as assessed by the Y-maze may be more acutely sensitive to early neuroplastic changes, which are maximized at shorter irradiation times, whereas the benefits of longer PBMT durations may manifest in other cognitive domains not captured by this test.

Building upon this foundation, our study provides several novel insights that extend the existing body of knowledge. Specifically, we employed a partially invasive intracranial 660 nm LED approach, ensuring sufficient hippocampal exposure despite the limited penetration depth of red light. This methodological refinement is important, as it allowed us to directly evaluate the therapeutic efficacy of 660 nm PBMT under controlled delivery conditions. Furthermore, we performed a detailed region-specific analysis of hippocampal subfields, including CA1, CA2, CA3, and DG. Notably, PBMT produced more pronounced reductions of amyloid burden in CA1 and CA2, while DG exhibited a comparatively weaker response. Such differential vulnerability and responsiveness among hippocampal subregions have not been systematically emphasized in previous PBMT research, and our findings highlight the importance of considering subregional heterogeneity in therapeutic evaluations. Additionally, our experimental paradigm incorporated a prolonged intervention strategy, with PBMT administered once daily for 14 consecutive days, which enabled us to assess the cumulative benefits of sustained treatment compared with shorter regimens used in prior studies. Taken together, these aspects—treatment timing, delivery method, hippocampal subregion specificity, and intervention duration—provide new insight into how PBMT can be optimized to achieve maximal therapeutic benefits in Alzheimer’s disease models and lay important groundwork for translational efforts.

When considering translation of PBMT to clinical trials, several limitations and safety concerns should be acknowledged. Anatomical differences between rodents and humans, including greater skull thickness and scattering properties, may significantly reduce intracranial light penetration and necessitate modified delivery strategies. In addition, the heterogeneity of Alzheimer’s disease in human populations, encompassing differences in disease stage, genetic background, and comorbidities, may influence treatment responsiveness. While our study did not reveal any signs of thermal damage or inflammation, prolonged or repeated light exposure carries the potential risk of cumulative phototoxicity or tissue stress that requires careful evaluation. Therefore, future preclinical and clinical investigations should prioritize systematic safety assessments and parameter optimization to ensure that PBMT achieves therapeutic efficacy without compromising long-term biological safety.

However, this study presents several limitations that not only restrict the generalizability of our findings but also point to key avenues for future investigation. The reliance on a single transgenic model that, while recapitulating amyloid pathology, lacks key features such as tau aggregation and late-stage neurodegeneration, limiting broader applicability. Moreover, although PBMT was shown to upregulate SIRT1 expression, the precise molecular mechanisms underlying its impact on Aβ clearance and neuronal resilience remain insufficiently characterized, necessitating in-depth molecular analyses. Additionally, we did not perform correlation analyses between individual behavioral outcomes and immunohistochemical markers; future studies should investigate whether PBMT-induced changes in Aβ plaque load and SIRT1 expression directly correlate with improvements in memory performance. Finally, behavioral assessments were restricted to the Y-maze test, which primarily evaluates working memory and does not capture other domains such as spatial learning, long-term memory, or executive function. Future studies should therefore employ multiple AD models, conduct multi-dimensional behavioral testing (e.g., Morris water maze, novel object recognition etc.), to provide a more comprehensive assessment of memory and to validate the cognitive benefits of PBMT observed in our study.

## 4. Materials and Methods

### 4.1. Animals

6~7-week-old 5xFAD male mice were purchased from Jackson Laboratory (Bar Harbor, ME, USA) to develop AD mouse model and underwent an acclimatization period of at least one week to adapt to the laboratory environment prior to experimentation. The 5xFAD male mice exhibit overexpression of mutant human APP699 gene, carrying Familial AD mutations K670N and M671L (Swedish), I716V (Florida), and V717I (London), along with human PS1 that includes two familial AD mutations, M146L and L286V [29]. The mouse Thy1 promoter regulates these transgenes, leading to their elevated expression in the brain [49]. Age-matched male C57BL/6J mice were used as normal control. 5xFAD mice were divided into three groups (n = 10) according to the presence and duration of photobiomodulation, with their data averaged for quantitative analysis.

After obtaining the mice, they were placed in an individual ventilation cage system (391W × 199D × 160H mm) and maintained in a controlled environment with a 12-h light/dark cycle at a temperature of 22 ± 1 °C, humidity of 50 ± 10%, and illumination range of 150–300 lux, with 10 to 15 ventilation cycles per hour. A commercial diet and water were available to the mice ad libitum. Every effort was made to minimize their suffering, and animal use was restricted to the smallest number needed for reliable scientific results.

This animal experiment was conducted by the Daegu-Gyeongbuk Advanced Medical Industry Promotion Foundation in compliance with the Animal Protection Act (enacted on 31 May 1991, Act No. 4379, and partially amended on 11 February 2020, Act No. 12053). The study was approved by the Institutional Animal Care and Use Committee (IACUC) of the Preclinical Center, with approval number KMEDI-2 2030801-00.

### 4.2. PBM Treatment Procedure Using LED Device

**Figure 6:**
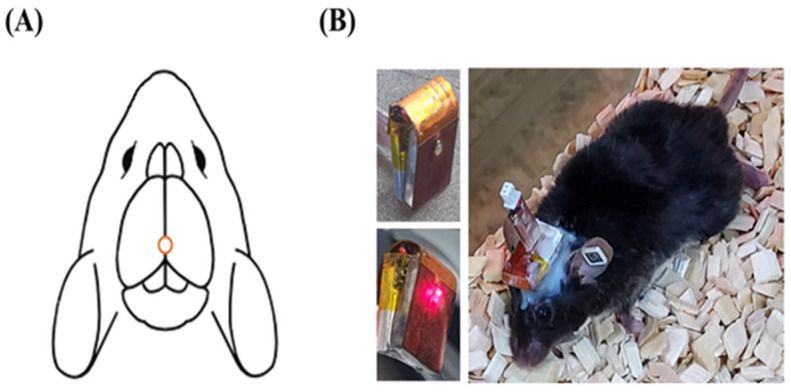
LED optical therapy device configuration and attachment in mouse. (**A**) Schematic of the drilling site on the mouse skull (−2.00 mm posterior to the bregma). (**B**) Photos of the LED optical therapy device, its light activation, and secured attachment on a mouse.

A stereotaxic instrument designed for small animals was used to secure the LED optical therapy device in place. To attach the device, the mouse scalp was fully removed, and a hole was drilled in the skull at approximately −2.00 mm posterior to the bregma to allow direct light irradiation into the brain. The LED lamp was then positioned in close contact with the skull and affixed using surgical cement, ensuring the eyes were not affected (Figure 6). Once stabilized, the device was powered appropriately, and an LED output of 7 mW was controlled using a wireless remote system. Table 1 represents all relevant parameters of the LED device. The light source was applied once daily for 30, 60, or 120 min, depending on the assigned experimental group, over a total period of 14 days. Although the LED produced no detectable heat on the implant or cranium, its temperature was further stabilized near room temperature using a standard cooling fan, which minimized the risk of thermal drift in the peak wavelength. To maintain consistent conditions, the treatment device was recharged every other day, and control and 5xFAD sham groups were experienced the same handling stress by connecting the charger without activating the LED.

### 4.3. Immunohistochemistry

Following PBM treatment, mice were anesthetized with 5% isoflurane (JW Pharm, Seoul, Republic of Korea) and transcardially perfused with 4% paraformaldehyde in 0.1 M phosphate buffer (pH 7.4, 4 °C). The brains were post-fixed overnight, followed by cryoprotection in 18% sucrose/PBS to ensure optimal tissue preservation. Using a cryostat, coronal brain sections (40 µm thick) encompassing the dorsal hippocampus were then obtained and subsequently preserved in an antifreeze solution at −20 °C for further immunohistochemical processing. Immunohistochemistry was conducted in accordance with the protocol described in our earlier study [41], beginning with fixation of hippocampus in 10% paraformaldehyde, followed by paraffin embedding. Paraffin blocks were then sectioned into thin slices of 4–6 μm and mounted onto glass slides. Deparaffinization was performed using xylene, followed by sequential dilutions in ethanol solutions, and hematoxylin–eosin (H&E) staining was used to assess tissue morphology. To inhibit endogenous peroxidase activity, tissue sections were treated overnight at room temperature with 0.3% hydrogen peroxide in methanol. Primary antibodies, diluted at ratios ranging from 1:1000 to 1:2000 in PBS containing 1% bovine serum albumin (BSA), were then applied and incubated overnight at 4 °C. The following day, HRP-conjugated secondary antibodies (1:500 in 5% BSA) were administered and incubated at 37 °C for 1 h. Additional staining was performed using 1% Schiff’s reagent in combination with Mayer’s hematoxylin, each applied for 5 min at room temperature.

### 4.4. Antibodies

Immunohistochemical analysis was conducted using specific primary antibodies; For β amyloid plaque immunostaining: rabbit anti-β amyloid 1-42 antibody, dilution 1:1000 (Product Code #ab201060, Abcam, Cambridge, UK); rabbit anti-β amyloid 1-40 antibody, dilution 1:1000 (Product Code #44-136, Invitrogen, Waltham, MA, USA). For SIRT1, mouse anti-SIRT1 antibody, dilution 1:2000 (Product Code #ab110304, Abcam, Toronto, ON, Canada). Mouse and rabbit IgG-HRP antibodies were used as secondary antibodies.

### 4.5. Image Acquisition and Quantification of Histology Data

The stained mice hippocampal sections were visualized and photographed with an equipped slide scanner (MoticEasyScan One, Schertz, TX 78154, USA) and image analysis was carried out with ImageJ software (version 1.54g, NIH, Bethesda, MD, USA). To ensure consistent intensity measurements, images were first converted to 8-bit grayscale. Subsequently, a specific threshold value was set to extract stained areas, ensuring consistency within the same batch of control and transgenic slices. The ImageJ software then calculated the area covered by positive staining. In this experiment, plaque load was quantified and expressed as the percentage of the total area occupied by pathology, following established methods [50]. Using ImageJ, an intensity threshold was set to selectively capture stained plaques while excluding background. The total plaque area from all analyzed region was summed and divided by the total hippocampal area to calculate the percentage of plaque load. This percentage was then averaged across mice within each experimental group. After that, the analyzed values were obtained and processed statistically.

### 4.6. Y-Maze Test for Behavioral Assessment

**Figure 7:**
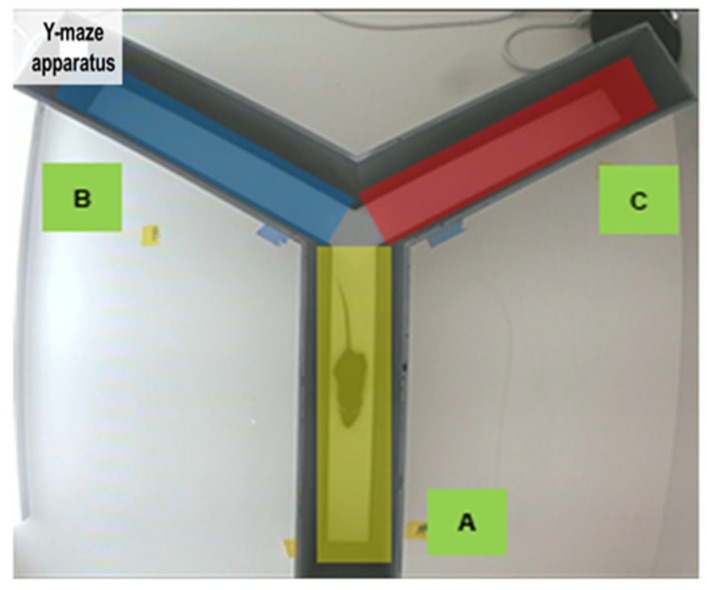
Y-maze test for cognitive ability behavioral experiment.

After PBM treatment using LED at 660 nm, hippocampus-dependent short-term spatial memory abilities were evaluated with the Y-maze test. Mice were placed at the end of one arm of a 120- degree Y-shaped maze, and the degree of arm alteration (e.g., ABC, BCA, CAB, CBA, etc.) was assessed by checking how much the mice moved to a new area in three arms (A, B, C) for 10 min (Figure 7). The main index for evaluating cognitive ability is the spontaneous alteration index, which reflects the tendency of the mouse to explore new arms rather than revisiting previously entered ones. Additionally, total distance traveled and the number of arm entries were recorded as indicators to check the basic motor ability status of the mouse. Mouse movements were continuously recorded on video and analyzed using a video-based tracking system for behavioral assessment.

### 4.7. Statistical Analysis

Quantified data are presented as the mean ± SEM. Significant differences between the groups were compared using Student’s t-test for independent means in Microsoft Excel. Statistical significance was set at *p* < 0.05 and *p* < 0.01.

## 5. Conclusions

The findings of this study demonstrate that prolonged early intervention with PBMT orchestrates a multifaceted neuroprotective response, including reduced amyloid-β accumulation, upregulated SIRT1 expression, and mitigation of spatial memory deficits. While these results highlight PBMT as a promising therapeutic strategy for attenuating Alzheimer’s disease progression, the beneficial effects were achieved using a partially invasive intracranial method, which necessitates caution in interpreting the translational potential. Further investigations are required to validate its efficacy and safety under fully non-invasive clinical conditions, optimize irradiation parameters such as wavelength, intensity, and duration, and determine the durability of its effects over extended treatment periods. Additional studies employing diverse AD models and multidimensional behavioral assessments will be important to confirm the generalizability of these findings and to clarify the mechanistic pathways underlying PBMT-mediated neuroprotection. Such efforts will ultimately determine whether PBMT can be refined into a clinically viable approach for preventing or slowing the progression of Alzheimer’s disease.

## Figures and Tables

**Table 1:** Irradiation parameters of LED device.

Parameters [Unit]	Value
Center wavelength [nm]	660
Output mode	Continuous
Average radiant power [mW]	7
Spot area [mm^2^]	1.28
Beam profile	Square
Beam divergence [°]	120

## Data Availability

Data are contained within the article.
